# A model system for studying plant–microbe interactions under snow

**DOI:** 10.1093/plphys/kiab027

**Published:** 2021-02-02

**Authors:** Chikako Kuwabara, Kentaro Sasaki, Natsuki Umeki, Tamotsu Hoshino, Wataru Saburi, Hirokazu Matsui, Ryozo Imai

**Affiliations:** 1 Hokkaido Agricultural Research Center, National Agriculture and Food Research Organization (NARO), Sapporo, Japan; 2 Division of Applied Genetics, Institute of Agrobiological Sciences, National Agriculture and Food Research Organization (NARO), Tsukuba, Japan; 3 Research Faculty of Agriculture, Hokkaido University, Sapporo, Japan; 4 Bioproduction Research Institute of Genome-based Biofactory, National Institute of Advanced Industrial Science and Technology (AIST), Sapporo, Japan

## Abstract

A model plant–pathogen system using Arabidopsis and its natural snow mold pathogen *Typhula ishikariensis* demonstrated Arabidopsis plants develop disease resistance through cold acclimation.

Dear Editor,

Overwintering plants must survive harsh winter conditions, including potentially lethal freezing temperatures. Snow cover, however, insulates and protects plants from extremely low temperatures in locations where snow is deep and persistent. Temperatures are typically maintained around 0°C, along with high humidity and darkness (Matsumoto, 2009). These conditions allow psychrophilic fungi, generally known as snow molds, to proliferate and infect plants (Hoshino et al., 2009). Snow mold diseases affect the production of winter wheat (*Triticum aestivum)*, rye (*Secale cereale*), and forage grasses in heavy snow regions. The major snow mold pathogens of winter cereals include *Typhula ishikariensis*, *Sclerotinia borealis*, and *Microdochium nivale* (Hoshino et al., 2009). Once snow melts, snow mold infections are visible in the fields as circular and irregularly shaped patches of gray, pink, or straw-color. The water-soaked, slimy leaves are typically covered with mycelia or sclerotia of the infecting pathogen (Hsiang et al., 1999).

Overwintering plants develop freezing tolerance when they are exposed to late autumn chilling temperatures and short days. The molecular, biochemical, and physiological changes involved in cold acclimation or winter hardening have been extensively studied (Thomashow, 1999). Some species of plants also undergo an increase in disease resistance during cold acclimation (Tronsmo, 1984; Kuwabara and Imai, 2009). Winter wheat exhibits an increase in resistance to pink snow mold (caused by *M. nivale*) after cold acclimation (Nakajima and Abe, 1996). In addition, barley (*Hordeum vulgare*) and meadow fescue (*Festuca pratensis*) acquire resistance to *Bipolaris sorokiniana* through cold acclimation (Płażek et al., 2003). Cold-acclimated (CA) plants express a variety of pathogenesis-related (PR) genes, and a subtractive hybridization study indicated that 17% of cold-induced genes encode PR proteins (Gaudet et al., 2003). Some cold-induced PR proteins exhibit anti-microbial activity in vitro (Kuwabara et al., 2002), which may indicate their role in cold-induced disease resistance. However, we lack a comprehensive understanding of how disease resistance is acquired through cold acclimation. Cold-induced defense mechanisms must be prophylactic and long-lasting, in contrast to the defense mechanisms induced by pathogen attack. A model host–pathogen system for studying cold-induced disease resistance and plant–microbe interactions under snow would greatly facilitate investigations at the molecular level.

Arabidopsis (*Arabidopsis thaliana*) has an overwintering growth habit and naturally undergoes cold acclimation and vernalization (Bond et al., 2011); here, we tested whether Arabidopsis could be used as a model system to study plant–microbe interactions under snow. We evaluated the overwintering response of several Arabidopsis ecotypes under heavy snow cover since adaptation to a winter environment varies with ecotype and reflects their geographical origin. The experiment was conducted at the NARO Hokkaido Agricultural Research Center, Sapporo (43°00′N, 141°25′E), Japan, from November 2006 to April 2007. A set of 18 Arabidopsis ecotypes were grown in pots in a greenhouse for 3 weeks and then CA for 3 weeks in an outdoor open-air space in which they were protected from snow by a roof. Pots were subsequently placed under snow cover when snow cover was persistent in the field (Figures 1, A and B) and left to overwinter until complete snow melt in April. Heavy damage occurred among several ecotypes. In the Eniwa ecotype (Hokkaido, Japan), two plants completely lost their rosette leaves (Figures 1, C and D), and some rosette leaves partially lost their original shape and were discolored (Figures 1, G and H), suggesting possible snow mold infection. Other ecotypes, including Col-0, appeared healthier with fewer damaged leaves (Figures 1, E and F). Discolored leaves were collected from all ecotypes and subjected to fungal isolation. Interestingly, three morphologically distinct fungi were obtained only from the leaves of the Eniwa ecotype. These were identified as *T. ishikariensis*, *T. incarnata*, and *Sclerotinia trifoliorum*, based on morphological characteristics, internal transcribed spacer sequencing, and mating patterns with tester species (Supplementary text). *Typhula ishikariensis* and *T. incarnata* isolates formed typical dark brown and brown sclerotia on  a potato dextrose agar (PDA) medium, respectively (Figures 1, I and J) and exhibited hyphal clamp connections (Figures 1, K and L). The isolates were designated as *T. ishikariensis* WSL9-5 and *T. incarnata* WSL9-1 and were used in subsequent studies.

**Figure 1:**
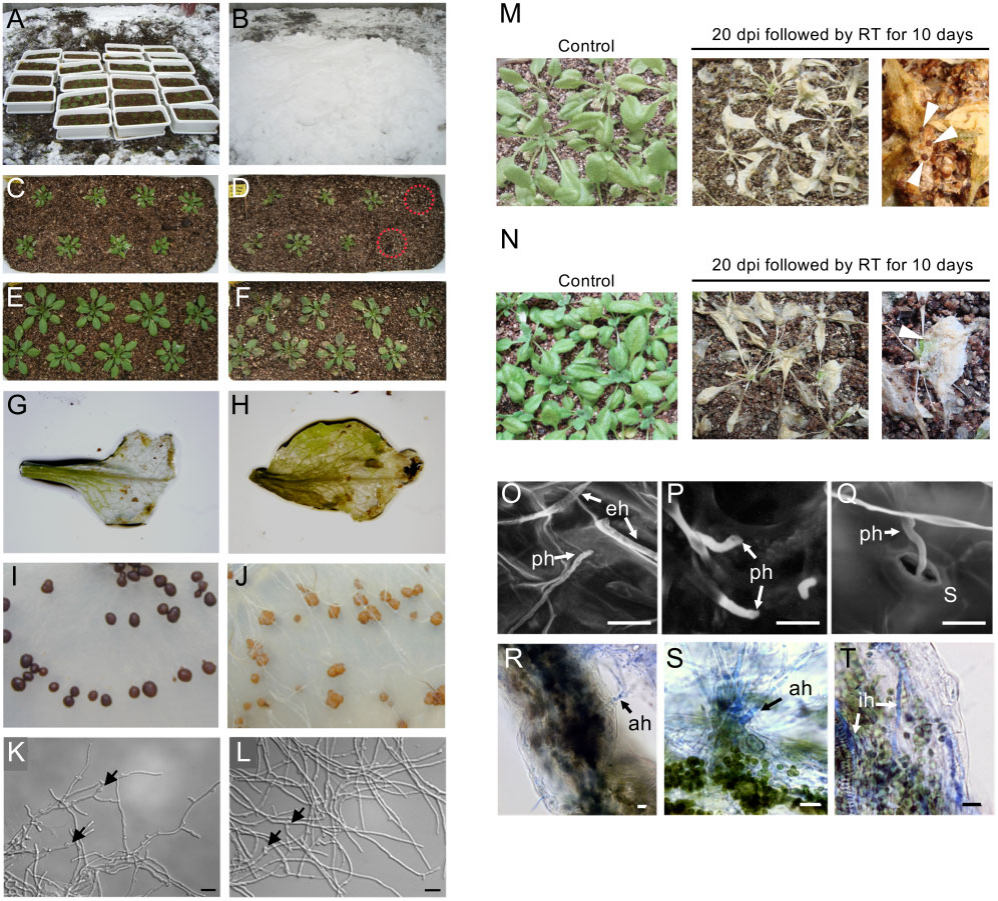
Isolation of snow mold fungi from overwintering Arabidopsis and their pathogenicity on Arabidopsis. A and B, Eighteen ecotypes of Arabidopsis were evaluated for susceptibility to snow mold. Three-week-old, CA Arabidopsis plants were placed in the field (A) and covered with snow on 18 December 2006 (B). Plants remained under snow cover until April when snow had completely melted. C and D, Eniwa ecotype prior to being covered in snow (C) and after snow melt in April (D). E and F, Col-0 ecotype prior to being covered in snow (E) and after snow melt in April (F). G and H, Rosette leaves of the Eniwa ecotype with snow mold-like symptoms, water-soaking, decay, and the formation of transparent lesions. I and J, Growth of isolated *T. ishikariensis* (I) and *T. incarnata* (J) strains on PDA medium after one month at 4°C. K and L, Microscopic observations of mycelia of *T. ishikariensis* (K) and *T. incarnata* (L). Clamp connections, indicated by arrows, are a unique characteristic of *Typhula* species. Scale bars, 20 µm. M and N, Wheat bran cultures of *T. ishikariensis* WSL9-5 (M) and *T. incarnata* WSL9-1 (N) were dispersed on the potted soil surface of soil-grown Arabidopsis and plants were maintained at 3°C under high relative humidity. Plants were transferred to room temperature at 20 dpi. Straw-colored leaves, similar to symptoms that occur in winter cereals infected with snow mold in the field, were observed in the inoculated Arabidopsis plants. Sclerotia on the leaf surfaces are indicated by white arrowheads. RT, room temperature. O-Q, SEM images of *T. ishikariensis* WSL9-5 mycelia on the epidermal surface of inoculated Arabidopsis leaves. R-T, Light micrographs of penetration sites in leaves infected with *T. ishikariensis* WSL9-5. A transverse section of a leaf exhibiting direct penetration by fungal mycelia. ph, penetrated hyphae; eh, external hyphae; S, stomata; ah, aggregated hyphae; ih, internal hyphae. Scale bars, 10 µm.

We first determined if *T. ishikariensis* WSL9-5 and *T. incarnata* WSL9-1 could infect the Arabidopsis Col-0 ecotype under laboratory conditions. Wheat-bran cultures of snow mold isolates were scattered on the soil surface around 4-week-old Col-0 plants, and the plants were kept at 3°C under dark and humid conditions that mimicked a snow-covered environment. After 20 d at 3°C, followed by 10 d at room temperature, leaves of inoculated plants exhibited discoloration symptoms, similar to snow mold-infected cereal leaves after snow melt under field conditions (Figures 1, M and N). Sclerotia were observed on the symptomatic, straw-colored leaves (Figures 1, M and N). Thus, snow mold isolates of *T. ishikariensis* and *T. incarnata* can infect Col-0 under controlled, laboratory conditions.

A rosette leaf on Col-0 plants was inoculated with *T. ishikariensis* WSL9-5, and monitored for the development of symptoms to determine the mode of infection (Supplemental Figure S1). A water-soaked and transparent lesion was observed around the inoculation site at 33-d post inoculation (dpi), and the lesion was subsequently enlarged by 41 dpi (Supplemental Figure S1). The inoculated leaf was partially rotted by 62 dpi and symptoms had spread to adjacent leaves. The symptoms were observed in all leaves of the plant by 77 dpi. Examination of the inoculated Arabidopsis leaf surface using  scanning electron microscopy (SEM) and light microscopy revealed that mycelia of WSL9-5 was clearly growing over the leaf surface (Figures 1, O–T). SEM further revealed that the initial invasion of hyphae into plants occurred by penetration of the cuticle (Figures 1, O and P) and stomata (Figure 1Q). This observation is similar to previous reports for snow mold infection of bentgrass (*Agrostis stolonifera*) and wheat (Oshiman et al., 1995). Stomatal entry was less frequent than cuticle penetration. Light microscopic observations revealed that hyphae sometimes aggregated and penetrated the cuticle layer, and infection hyphae grew into the internal spaces of the leaves (Figures 1, R–T).

A quantitative assay system for pathogenicity and host resistance is required to investigate the host–microbe interactions at a molecular level. A detached leaf assay to evaluate snow mold resistance in barley has been previously reported (Watanabe et al., 2003). We developed a modification of this method that provides a simple and consistent quantitative evaluation using detached leaves of Arabidopsis. Briefly, an agar plug containing snow mold mycelia of WSL9-5 was placed at the center of a detached rosette leaf, and the leaves with the plug were placed on wet filter paper in a petri dish and incubated at 4°C under dark and humid conditions, mimicking the environment in which snow mold infections naturally occur. The petri dish containing inoculated leaves was returned to room temperature after 10 d and the lesioned areas (mm^2^) were measured 2 d later.

The pathogenicity of WSL9-5 and other *T. ishikariensis* isolates belonging to biotype A (biological species I) and biotype B (biological species II; Matsumoto et al., 1982) was then compared to evaluate the specificity of the assay system. Lesions developed on leaves inoculated with WSL9-5 (biotype A) and the three biotype A isolates from other plant species (MAFF306133, MAFF306134, PR750D). The lesion area caused by WSL9-5 was significantly larger than the lesions caused by the other biotype A isolates (Figures 2, A and B), suggesting that the virulence of WSL9-5 in Arabidopsis is stronger than that of the other biotype A isolates. In contrast, *T. ishikariensis* biotype B (TB36, MAFF306141) did not form lesions on leaves (Figures 2, A and B), which is consistent with the previous reports indicating that the biotype B only causes infections in monocots (Hoshino et al., 2009).

**Figure 2:**
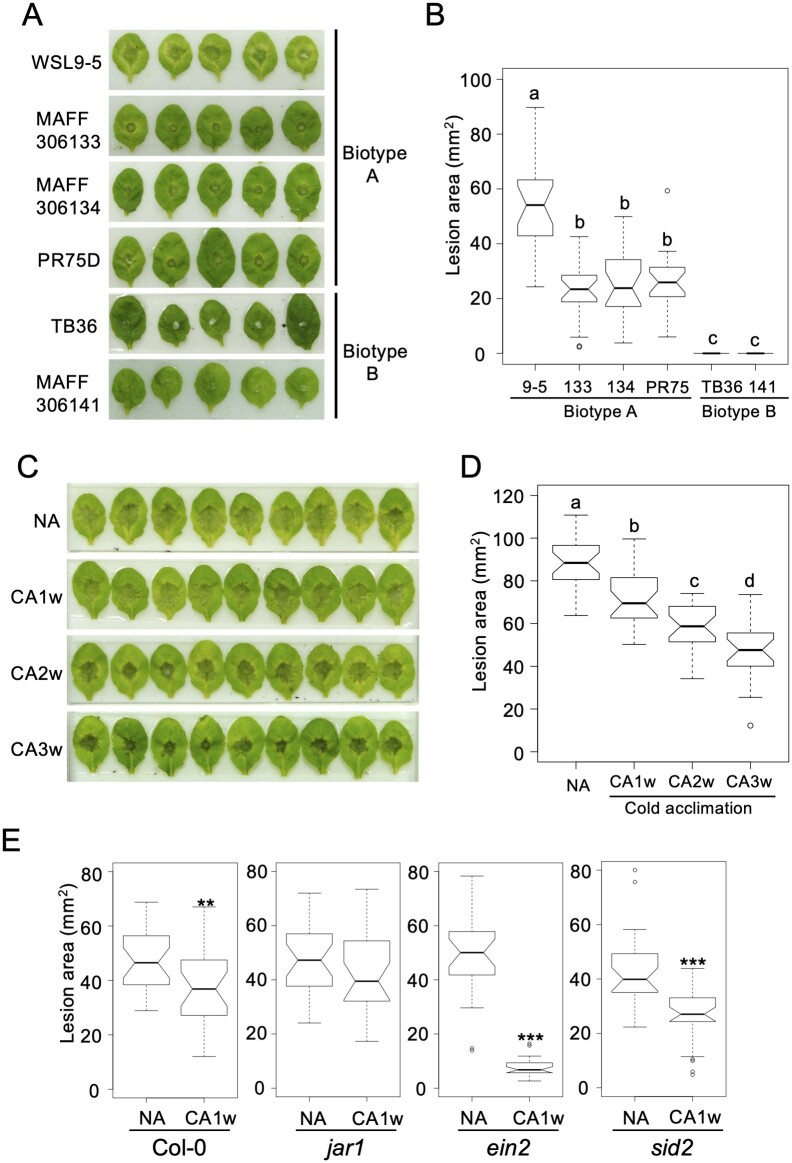
An Arabidopsis-*Typhula* assay system for investigating *T. ishikariensis* interactions with Arabidopsis. A, Representative disease symptoms on Arabidopsis leaves inoculated with *T. ishikariensis* WSL9-5 and other *T. ishikariensis* isolates of biotype A and B. B, Lesion area on Arabidopsis leaves inoculated with *T. ishikariensis* WSL9-5 and other *T. ishikariensis* isolates (*n* = 21, biological replicates). C, Representative disease symptoms on NA and CA Arabidopsis leaves caused by *T. ishikariensis* WSL9-5. Cold acclimation was achieved by transferring plants to 4°C (10-h light/14-h dark) for 1–3 weeks (CA1w to CA3w). D, Effect of cold acclimation on lesion area in Arabidopsis leaves caused by *T. ishikariensis* WSL9-5 (*n* = 23–27, biological replicates). For B and D, different letters indicate statistically significant differences between means (*P *<* *0.05, one-way analysis of variation (ANOVA) followed by Tukey–Kramer multiple comparisons test). E, Lesion area on the leaves of Arabidopsis mutants, *jar1*, *ein2*, and *sid2* with or without cold acclimation (4°C, 1 week) caused by *T. ishikariensis* WSL9-5 (*n* = 25–27, biological replicates). Significant differences determined relative to the NA leaves. **P <* *0.01 and ***P <* *0.001 based on Student’s *t* test. The box describes the 25th and 75th percentile with the median (50th percentile), and whiskers extend to 1.5 times the interquartile range. The notch represents the 95% confidence interval around the median. If the notches of two boxes do not overlap, there is strong evidence that their medians are significantly different. Outlying data points are represented as circles.

Overwintering plants, such as wheat and grasses, increase disease resistance when they undergo cold acclimation (Kuwabara and Imai, 2009). Therefore, leaves of CA and nonacclimated (NA) Arabidopsis plants were inoculated with WSL9-5 to determine if cold acclimation induces snow mold resistance in Arabidopsis. Leaves exhibited reduced lesion areas as the period and environmental conditions of cold acclimation were extended (Figures 2, C and D). Thus, it appears that cold acclimation increases snow mold resistance in Arabidopsis.

To perform initial characterization of the molecular mechanisms responsible for cold-induced disease resistance, Arabidopsis plants defective in different hormone signaling pathways were analyzed. Arabidopsis mutants, *jasmonate-resistance 1* (*jar1*), *ethylene-insensitive 2* (*ein2*), and *salicylic acid induction-deficient 2* (*sid2*) were CA and then inoculated with WSL9-5 for evaluation of disease resistance under the mimicked snow-covered condition (Alonso et al., 1999; Wildermuth et al., 2001; Staswick et al., 2002). Interestingly, the *jar1* mutant showed similar size legions with or without cold acclimation, indicating a lack of cold-induced disease resistance (Figure 2E). These data suggested that jasmonic acid may be involved in the disease resistance acquired through cold acclimation. In contrast, lesion areas on *ein2* and *sid2* leaves were reduced when cold-acclimation was applied, suggesting that these mutants retain the cold-induced defense mechanism (Figure 2E). The *ein2* mutant was of particular interest because CA *ein2* showed much smaller lesion areas than CA Col-0 (Figure 2E). This may suggest ethylene is a negative regulator of disease resistance acquired through cold acclimation. Since Jasmonic acid and ethylene are known as a positive and a negative regulator of freezing tolerance, respectively (Shi et al., 2012; Hu et al., 2013), cold-induced freezing tolerance and disease resistance can be highly linked through these hormones.

In our present study, we successfully isolated two *Typhula* snow molds that exhibit a significant level of virulence in Arabidopsis. *Typhula ishikariensis* WSL9-5 infects Arabidopsis primarily through epidermal penetration, and slowly spreads into the entire plant causing symptoms similar to those observed in cereals. We developed a detached-leaf inoculation method that enabled a quantitative assessment of pathogenicity and plant resistance in a short period of time. The Arabidopsis model system will be useful for addressing questions on plant–snow mold interactions that occur when plants are covered by snow, including how does cold acclimation induce plant resistance, is the resistance signaling pathway common to freezing tolerance induction, how do host and pathogen genotypes and physiology affect the pathogenicity and disease development of snow molds, and how do environmental factors, such as nonfreezing low temperature, darkness, or humidity, affect disease development? Since these research topics have been barely examined, addressing these questions will contribute to the timely development of snow mold resistant cultivars in cereals and forage crops. 

## Supplemental data

**Supplemental Figure S1.** Whole-plant leaf inoculation assay.

**Supplemental Materials and Methods.**

**Supplemental text**. Descriptions of isolated snow mold fungi.

## Supplementary Material

kiab027_Supplementary_Data

## Notes

R.I. conceived the idea for the study; K.S. isolated snow molds; T.H. identified snow mold isolates; C.K. conducted pathogenicity tests and developed inoculation assays; C.K., K.S., and N.U. conducted the detached leaf inoculation assay; K.S., W.S., H.M., and R.I analyzed data; C.K., K.S., and R.I. wrote the manuscript.

The author responsible for distribution of materials integral to the findings presented in this article in accordance with the policy described in the Instructions for Authors (https://academic.oup.com/plphys/pages/general-instructions) is: Ryozo Imai (rzi@affrc.go.jp).
