# Supplementary material for: A model system for studying plant–microbe interactions under snow
Source: Plant Physiol. 2021 Feb 2;185(4):1489–94. doi: 10.1093/plphys/kiab027 (PMC8133538; doi:10.1093/plphys/kiab027)
Supplement: kiab027_Supplementary_Data [file kiab027_supplementary_data.zip › pp.01579.2020-s01.pdf]

Supplemental Materials and Methods

Plant material.

Seeds of Arabidopsis were surface sterilized for 30 min in 70% ethanol containing 0.02% Tween20 and washed for 1min in 100% ethanol. The sterilized seeds were dried on sterilized filter paper and then sown on Murashige and Skoog (MS) agar medium. Seeds sown on MS medium were left for 2 days at 4 °C for stratification. Plants were then incubated in a growth chamber at 22˚C under long day (16 h light / 8 h dark) conditions. Arabidopsis seedlings were transferred to pots containing sterilized soil 10-12 d after germination.

Overwintering of Arabidopsis ecotypes under snow cover.

Plants of eighteen Arabidopsis ecotypes derived from various regions were overwintered under field conditions to isolate snow molds that had infected the Arabidopsis plants. The Arabidopsis ecotypes were; Col-0 (standard natural accession), Sap-0 (Hokkaido, Japan), Sap-1 (Hokkaido, Japan), Sap-2 (Hokkaido, Japan), Eniwa (Hokkaido, Japan), TKS (Tokushima, Japan), OY (Okayama, Japan), Fuk (Fukuyama, Japan), ES (Enoshima, Japan), Ge-2 (Switzerland), Zu-0 (Switzerland), Ka-0 (Austria), Pi-0 (Austria), Mv-0 (USA), Sf-0 (Spain), Sf-1 (Spain), Sf-2 (Spain), Can and (Canary Islands). Seeds were obtained from the RIKEN BioResource Center (https://en.brc.riken.jp). The ecotypes were grown on MS agar media and then transferred to plastic containers filled with soil. Plants were grown in a greenhouse for 3 weeks and subsequently moved to an outdoor space on November 27, 2006 where a roof cover prevented snow from covering the plants. After three weeks of outdoor acclimation, snow was removed from uncovered field location and the containers with plants were placed atop field soil on December 18, 2006. Immediately afterwards, the removed snow was then used to completely cover all plants with snow pack. Plants overwintered under the snow until the beginning of April.

Plants were examined for disease symptoms on April 8, 2007 when snow cover had completely disappeared.

Isolation of snow molds from Arabidopsis.

Rosette leaves of Arabidopsis ecotypes with symptoms of snow mold infection were collected in plastic bags and kept at 4°C prior to fungal isolation. The collected leaves were surface-sterilized with 70% (v/v) ethanol and then thoroughly washed with sterilized distilled water and placed on potato dextrose agar (PDA; Becton, Dickinson and Company) at 4°C. Mycelia from the growing margins of developing colonies were transferred to new PDA plates.

ITS sequence analysis of fungal isolates.

Fungal isolates were cultured for 1 month at 10˚C on PDA. All isolates produced sclerotia, which were harvested (0.1 g per isolate) and used for DNA extraction with a ISOPLANT II (Nippon Gene, Japan) kit. The ITS region of genomic rDNA was amplified using the primer pair ITS1 (5'-TCCGTAGGTGAACCTGCGG) and ITS4 (5'-TCCTCCGCTTATTGATATGC), as previously described (Hsiang and Wu, 2000). The PCR product was purified using a QIAquick PCR Purification Kit (QIAGEN) and sequenced on an ABI PRISM 3100 Genetic Analyzer (Applied Biosystems) using the ITS1 primer. ITS gene sequences of related taxa were obtained from the DNA Data Bank of Japan (DDBJ). Multiple alignments were performed using CLUSTALW via DDBJ.

Pathogenicity tests:

Pathogenicity of *T. ishikariensis* WSL9-5 and *T. incarnata* WSL9-1 was evaluated in Arabidopsis Col-0 under laboratory conditions. Four-week-old plants grown under short day

conditions were used. *T. ishikariensis* WSL9-5 and *T. incarnata* WSL9-1 were grown on PDA agar plates at 10˚C and agar plugs (4 mm in diameter) containing hyphae were removed from 2-3 week-old cultures and transferred to an autoclaved mixture of wheat bran (mixture of 6 g vermiculite, 6 g wheat bran, and 12 ml of distilled water in a 100 ml flask) and incubated at 10˚C in the dark for approximately two weeks. Wheat bran inoculum (4.5 g) containing mycelia was dispersed on the surface of the soil in a pot (21 cm x 13 cm x 4 cm) with 20 plants. The plants were then placed in black plastic bags with a wet paper towel covering the plants to maintain a high relative humidity and incubated at 3˚C in the dark.

Whole plant leaf inoculation assay.

Four- to five-week-old Arabidopsis plants were used in the assay. *T. ishikariensis* WSL9-5 was cultured on PDA agar plates for 2-3 weeks at 4˚C in the dark. Agar plugs (2 mm in diameter) were removed from the edge of developing colonies to enrich the inoculum with tip-growing hyphae. An agar plug was placed in the center of one of the fully developed leaves of each plant. Inoculated plants were placed in a plastic bag with a wet paper towel covering them to maintain a high relative humidity, and incubated at -0.2˚C in the dark.

Microscopic observations of infected leaves.

Inoculated leaves were hand-sectioned and stained with an aniline blue solution (15% (v/v) phenol, 15% (v/v) acetic acid, 30% (v/v) glycerol, and 0.025% (w/v) aniline blue) for 5 min at seven days after inoculation. Stained sections of leaf samples were mounted in 10% glycerol and examined using a FW4000 light microscope (Leica). Surface features of the inoculated Arabidopsis leaves were also prepared for and observed by scanning electron microscopy (SEM) using a JSM-T330A SEM (JEOL, Japan) operating at a 10 kV accelerating voltage.

Detached leaf inoculation assay.

Rosette leaves were cut and removed from 3-4 week-old Arabidopsis plants and placed on a wet filter paper in a petri dish. The center of each leaf was wounded with a sterile micropipette (yellow) tip. A hyphal plug, prepared as described above, was then placed over the wound site of each detached leaf. The petri dishes were sealed with parafilm to maintain a high relative humidity and wrapped in aluminum foil to ensure dark conditions and incubated at 4˚C. The petri dishes were transferred to room temperature after 10 d, and under light for 2 d. Lesion area was calculated using digital images of the leaves processed in Image J software (https://imagej.nih.gov/ij/download.html). Boxplots were drawn in R using RStudio (v1.2.5033).

LITERATURE CITED

Hsiang T, Wu C (2000) Genetic relationships of pathogenic *Typhula* species assessed by RAPD, ITS-RFLP and ITS sequencing. Mycol Res **104**: 16–22
