# Supplementary material for: A model system for studying plant–microbe interactions under snow
Source: Plant Physiol. 2021 Feb 2;185(4):1489–94. doi: 10.1093/plphys/kiab027 (PMC8133538; doi:10.1093/plphys/kiab027)
Supplement: kiab027_Supplementary_Data [file kiab027_supplementary_data.zip › pp.01579.2020-s02.pdf]

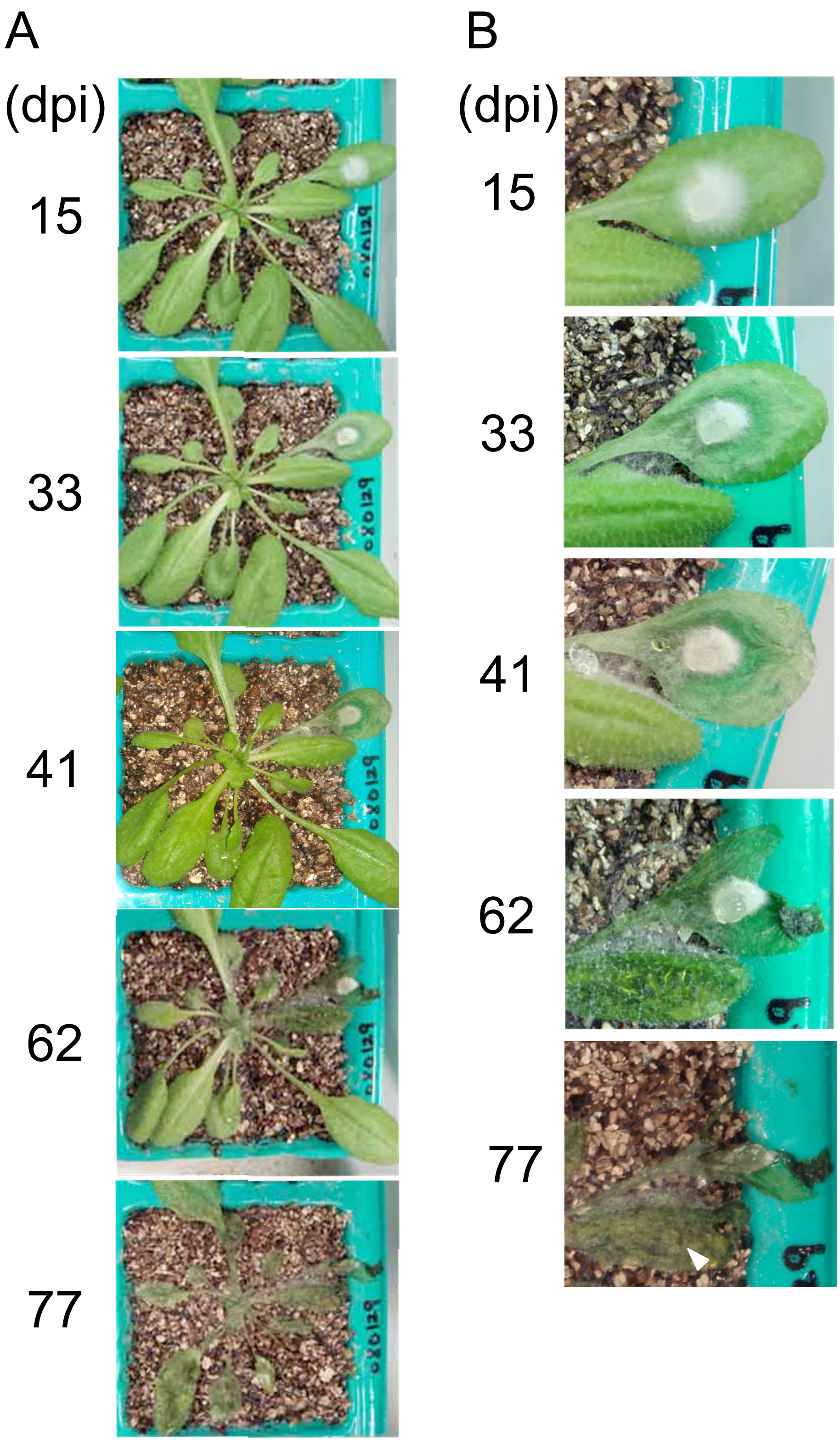

Supplemental Fig. S1 Whole plant leaf inoculation assay. A, Disease progression was shown from days 15 to 77 after inoculation of mycelial agar plug (at -0.2 ˚C in a dark with high humidity). Disease symptoms spread from the inoculated leaf to adjacent leaves and to whole plant body at 77 days post inoculation (dpi). The width of the pots is 4 cm. B, Close-up photos of the inoculated leaf shown in panel A. The water-soaked lesion expanded concentrically from the inoculum. The infected leaf eventually collapsed at 62 dpi.
