# Supplementary material for: A model system for studying plant–microbe interactions under snow
Source: Plant Physiol. 2021 Feb 2;185(4):1489–94. doi: 10.1093/plphys/kiab027 (PMC8133538; doi:10.1093/plphys/kiab027)
Supplement: kiab027_Supplementary_Data [file kiab027_supplementary_data.zip › pp.01579.2020-s03.pdf]

Supplementary text

Descriptions of isolated snow mold fungi

Typhula ishikariensis WSL9-5

This isolate exhibits clamp connections in mycelia, forms white colonies, and brown (young) to dark brown (mature) spherical sclerotia (0.5-2 × 0.5-2.5 mm). Rind cell patterns in mature sclerotia are not lobate. Maximum mycelial growth of isolates on PDA was at 10°C, and no mycelial growth occurred at 20°C. Internal transcribed spacer (ITS) gene sequence of the isolate had high homology (97%) with *Typhula ishikariensis* biological species I[1,2]. *T. ishikariensis* is a well-known, psychrophilic snow mold of overwintering hay grasses of monocots, and dicots such as rape (*Brassica campestris* L.) [3]. The isolate (dikaryon also mates with tester monokaryons of *T. ishikariensis* biotype A (biological species I) from Hokkaido, Japan and does not mate with tester monokaryons of biotype B (biological species II). This suggests that the isolate obtained from infected Arabidopsis has the same genetic background as *T. ishikariensis* biotype A in Hokkaido, Japan. The isolate was identified as *T. ishikariensis* biotype A based on morphological and genetic characteristics and its ITS sequence. The isolate was designated as *T. ishikariensis* WSL9-5.

Typhula incarnata WSL9-1

The isolate exhibits clamp connections in mycelia and forms white to pale pinkish colonies and pinkish (young) to light brown (mature) irregular-shaped sclerotia (1-3 × 1-3 mm). Rind cell patterns in mature sclerotia are irregular and contain lobate patterns that can be described as being like interlocking jigsaw puzzle pieces. Maximum mycelial growth of isolates on PDA was at 10°C, while very little growth occurred at 20°C. ITS gene sequences of isolates had high homology (95-99%) with *Typhula incarnata*. This fungus is a well-known, psychrotrophic snow mold of overwintering hay grasses of monocots and can also attack overwintering dicots such as species in the Brassicaceae. All of the isolates (dikaryons) mated with the tester monokaryon of *T. incarnata* from Hokkaido, Japan. Isolates were identified as *T. incarnata* based on biological characteristics and its ITS sequence. The isolate was designated. *T. incarnata* WSL9-1.

Sclerotinia trifoliorum

These isolates did not exhibit clamp connections in their mycelia and they formed whitish colonies. Black-colored sclerotia (3-5 × 7-10 mm), which were similar in appearance to rat feces, were located on the surfaces of colonies and primarily near the edges of the PDA plates. Mature sclerotia could be easily separated from underlying colonies. Maximum mycelial growth of isolates on PDA was at 15-20°C, while very little growth occurred at 35°C. ITS gene sequences of isolates had high homology (>99%) with *Sclerotinia trifoliorum*. This fungus is a psychrotrophic snow mold of overwintering legumes and can attack other dicots, including one of the Brassicaceae, bittercress (*Cardamine flexuosa* With.)[4]. Isolates were identified as *S. trifoliorum* based on biological characteristics and their ITS sequence.

1. Hoshino, T., Xiao, N. & Tkachenko, O. B. Cold adaptation in the phytopathogenic fungi causing snow molds. *Mycoscience* **50**, 26–38 (2009).
2. Takemori, T. & Tomiyama, K. Effect of continuous cropping on overwintering crops (preliminary results). *Annu. Rep. Soc. Plant Prot. North Japan* **7**, 53–54 (1956).
3. Watanabe, T., Namikawa, Y., Saito, I. & Takasawa, T. Polygalacturonase activity produced by alfalfa culture of the psychrotrophic facultative snow mold *Sclerotinia triofoliorum* (in Japanese). *Res. Bull. Obihiro Univ.* **25**, 15–21 (2004).
4. Watanabe, T., Namikawa, Y., Saito, I. & Takasawa, T. Polygalacturonase activity produced by alfalfa culture of the psychrotrophic facultative snow mold *Sclerotinia triofoliorum* (in Japanese). Res. Bull. Obihiro Univ. 25, 15–21 (2004).
